# MRI-to-PET synthesis via deep learning for amyloid-β quantification in Alzheimer’s disease

**DOI:** 10.1007/s00330-025-12251-3

**Published:** 2026-01-07

**Authors:** Zhigeng Chen, Sheng Bi, Yi Shan, Feng Wang, Yong Wang, Zhongyuan Qi, Tao Wang, Xiaoyuan Li, Shengnan Li, Huanhui Xiao, Silun Wang, Bixiao Cui, Zhigang Qi, Ying Han, Shaozhen Yan, Jie Lu

**Affiliations:** 1 Department of Radiology and Nuclear Medicine, Xuanwu Hospital, Capital Medical University, Beijing, China; 2 Beijing Key Laboratory of Magnetic Resonance Imaging and Brain Informatics, Beijing, China; 3 Key Laboratory of Neurodegenerative Diseases, Ministry of Education, Beijing, China; 4 Department of Nuclear Medicine, Nanjing First Hospital, Nanjing Medical University, Nanjing, China; 5 Department of Radiology and Nuclear Medicine, The First Hospital of Hebei Medical University, Shijiazhuang, China; 6 YIWEI Medical Technology Co., Ltd., ShenZhen, China; 7 Department of Neurology, Xuanwu Hospital, Capital Medical University, Beijing, China

**Keywords:** Alzheimer’s disease, Amyloid-beta, Magnetic resonance imaging, Positron emission tomography, Generative adversarial network

## Abstract

**Objectives:**

Amyloid-β (Aβ) PET is crucial for diagnosing and monitoring Alzheimer’s disease (AD), but its high cost and radiation exposure limit its use. Deep learning techniques make it possible to generate PET from structured MRI data. In this study, we built a deep learning model to generate 3D synthetic Aβ PET images from structural MRI.

**Materials and methods:**

The generative adversarial network with share parameters (ShareGAN) model was trained and tested with 1009 Aβ PET and paired MRI images from the Alzheimer’s Disease Neuroimaging Initiative database and three tertiary hospitals in China. The 3D synthetic model operates on the whole volume rather than 2D image slices, realistically reproducing minor discrepancies between neighboring image planes. ShareGAN-based PET images were evaluated using quantitative metrics and visual assessment. Pearson correlation coefficient and Bland–Altman analyses were used to assess the correlation and concordance between synthetic and real PETs.

**Results:**

3D Synthetic PET images showed high similarity and correlation with real Aβ PET in external testing sets 1 and 2 in terms of structural similarity index measure (0.898, 0.899), peak signal-to-noise ratio (34.690, 34.725), mean absolute error (0.031, 0.031), and standardized uptake value ratio (R = 0.758, 0.828). The diagnostic accuracy of PET positive or negative status in external testing sets 1 and 2 was 88.5% and 89.4%, respectively.

**Conclusion:**

MRI-based 3D synthetic Aβ PET images can serve as a safe and cost-effective tool for Aβ status visualization, providing PET-eligible patients with Aβ PET-like imaging analysis to guide subsequent real Aβ PET scans.

**Key Points:**

***Question**** Amyloid-β (Aβ) PET limitations (high cost, radiation, limited access) hinder early Alzheimer’s disease (AD) detection. Clinical practice urgently requires a suitable supplementary method for Aβ pathology assessment*.

***Findings**** AI-synthesized 3D Synthetic Aβ PET from structural MRI demonstrated strong consistency with real PET and effectively triaged high-risk patients for confirmatory scans*.

***Clinical relevance**** This non-invasive, cost-effective method holds the promise of enabling wider Aβ pathology screening, reduces unnecessary PET scans, and supports early intervention in resource-limited settings, while preserving diagnostic rigor for treatment decisions*.

**Graphical Abstract:**

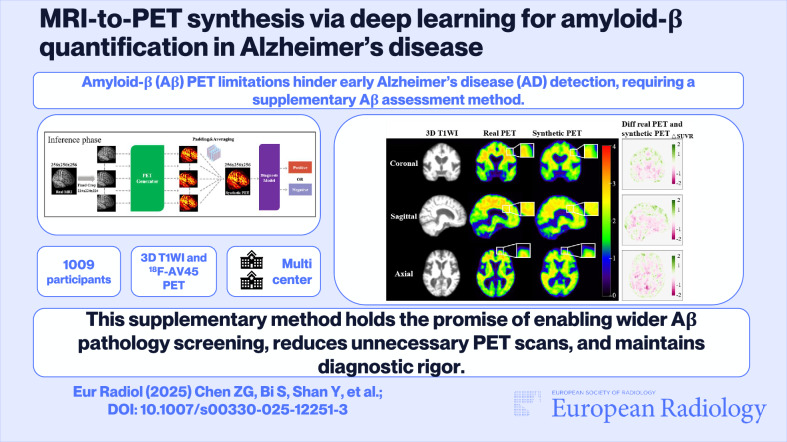

## Introduction

Alzheimer’s disease (AD) is characterized by the accumulation of amyloid-β (Aβ) plaques, phosphorylated tau protein, and progressive neurodegeneration [1]. Aβ deposition, detectable 14–18 years before diagnosis, drives neuronal damage and clinical symptoms [2–4]. Consequently, the development of reliable, accessible, and non-invasive methods for detecting and monitoring cerebral Aβ pathology in vivo is paramount for advancing early diagnosis, risk stratification, and evaluating the efficacy of emerging disease-modifying therapies. Available methods for assessing Aβ burden include cerebrospinal fluid (CSF) analysis, plasma biomarkers, and PET imaging [5]. However, this remains a significant clinical challenge due to limitations inherent in current approaches for assessing Aβ burden.

CSF biomarkers, such as CSF Aβ_42/40,_ provide high diagnostic accuracy and are widely used to reflect Aβ deposition in AD [6]. But CSF analysis requires an invasive lumbar puncture and may not fully reflect regional brain Aβ levels. Plasma-based Aβ assays have emerged as a more convenient and cost-effective option. Recent advances in the plasma p-tau217/Aβ42 ratio have shown promise for the accurate diagnosis of AD [7, 8]. However, due to limited cross-platform standardization, plasma biomarkers are not yet suitable for standalone diagnosis and still require confirmation using other modalities, such as PET [9]. Aβ PET imaging enables in vivo visualization of Aβ pathology, correlating strongly with neuropathology. An autopsy study confirmed that Aβ PET standardized uptake value ratio (SUVR) correlated highly with Aβ neuropathological changes [10]. An early diagnostic study showed that Aβ PET diagnosed AD with an area under the curve of 0.93 and differentiated progressive and stable mild cognitive impairment (MCI) with an area under the curve of 0.83 [11]. In current AD treatment monitoring and efficacy assessment, quantitative index of Aβ PET in specific brain regions is a key component of various metrics [12–14]. Despite its clinical value, it is hindered by high costs, limited accessibility of equipment, radiation exposure, and the logistical challenge of producing short-lived radiotracers ^18^F-florbetapir (^18^F-AV45).

Structural MRI is routinely used to assess brain structures such as cortical thickness and gray matter volume [15, 16]. Previous studies have revealed associations between regional Aβ deposition and cortical atrophy [17–19]. When Aβ deposition exceeds the compensatory capacity of the aging brain, it leads to accelerated cortical atrophy [20, 21]. A recent machine learning study integrating MRI with apolipoprotein E (APOE) and clinical data improved AD prediction (area under the curve from 0.84 to 0.89) [22]. Nevertheless, most studies provide only dichotomous results for Aβ status, and visualizing Aβ distribution patterns is still required for accurate AD assessment. Generative adversarial network (GAN), a class of deep learning models capable of cross-modal image synthesis, has shown promise in translating structural MRI into PET-like images [23–25]. In AD, GAN has predominantly been used to generate ^18^F-fluorodeoxyglucose (^18^F-FDG) PET-like images from MRI [25–28], whereas studies on Aβ PET synthesis remain relatively scarce. Additionally, the feasibility of quantitative analysis of Aβ PET images requires further investigation. Given the non-invasiveness, widespread availability, and cost-effectiveness of structural MRI, and the powerful cross-modal translation capabilities of GAN, we chose to combine these two modalities to synthesize Aβ PET images. Building upon the GAN model for MRI to ^18^F-FDG PET proposed by Wang et al [27], we introduced several adaptations to enable the generation of Aβ PET images from structural MRI and further explore the clinical applicability of the generated images in AD.

The aim of this study was to develop a GAN-based model for generating synthetic Aβ PET images from structural MRI, evaluate the similarity and quantitative consistency with real Aβ PET, and assess their potential as a supportive tool to identify patients who may require further confirmation with real PET scans.

## Materials and methods

### Study participants

This was a retrospective study approved by the Ethics Committee of Xuanwu Hospital, Capital Medical University, China. Written informed consent was given by all participants or their legal guardians before the start of the study.

**Fig. 1:**
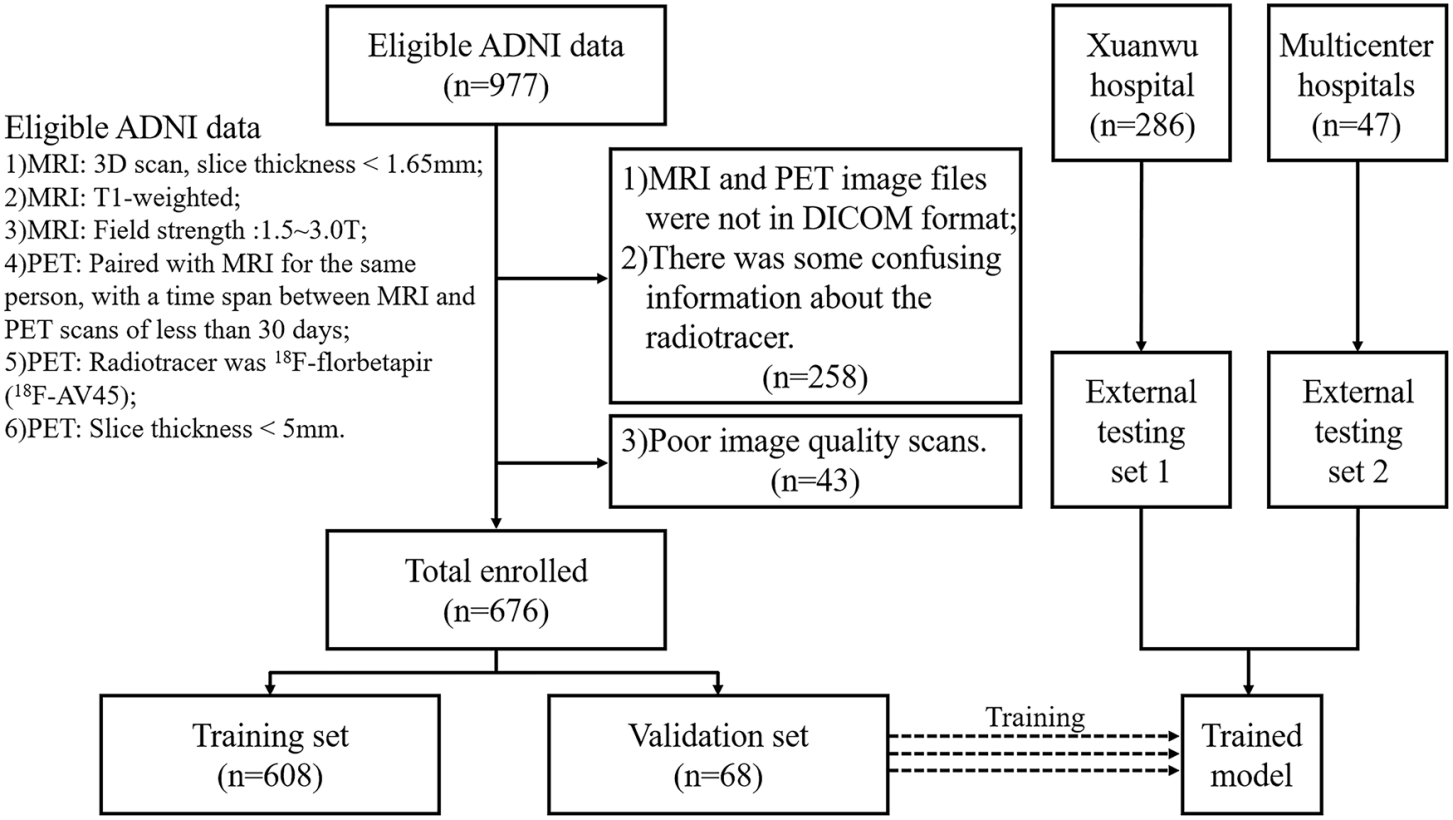
Flowchart of participant selection in this study. Multicenter hospitals consisted of Nanjing First Hospital, Nanjing Medical University and the First Hospital of Hebei Medical University. ADNI, Alzheimer’s Disease Neuroimaging Initiative; n, number; MRI, magnetic resonance imaging; PET, positron emission tomography; DICOM, Digital Imaging and Communications in Medicine

A total of 1009 subjects were included in this study, divided into training set, validation set, external testing set 1, and external testing set 2 (Fig. 1). The training set was used for model training, the validation set was used for model hyperparameter optimization, and external testing sets 1 and 2 were utilized for model performance evaluation.

The datasets of 676 participants in the Alzheimer’s Disease Neuroimaging Initiative (ADNI) database from April 2010 to January 2023 were collected. The dataset comprised the ^18^F-AV45 PET and paired high-resolution T1-weighted imaging (3D T1WI). The time span between MRI and PET scans was less than 30 days. We also collected the datasets of 286 participants at Xuanwu Hospital, Capital Medical University, from March 2023 to February 2024 as external testing set 1. The datasets of 32 participants and 15 participants were respectively obtained at Nanjing First Hospital from December 2022 to May 2024 and the First Hospital of Hebei Medical University from August 2023 to May 2024 used as external testing set 2.

All the enrolled data included healthy controls (HC), MCI, and AD, with data from Xuanwu Hospital (external testing set 1) also including vascular dementia (VaD), behavioral variant frontotemporal dementia (bvFTD), dementia with Lewy bodies (DLB), and semantic dementia (SD). More details of inclusion and exclusion criteria were given in Supplementary Material Sections 1 and 2.

The ^18^F-AV45 PET and 3D T1WI imaging data were acquired using the simultaneous PET/MR 3.0-Tesla system (uPMR 790, United Imaging Healthcare; Signa, GE Healthcare) and Siemens Biograph mCT PET scanner, Siemens Magnetom Prisma 3-T scanner. Acquisition parameters were detailed in Supplementary Material Section 3.

### Preprocessing

The tool package Statistical Parametric Mapping (SPM12, Wellcome Department of Imaging Neuroscience) in MATLAB (The MathWorks Inc.) was applied for preprocessing. The ^18^F-AV45 PET was registered to paired 3D T1WI, and then the MRI image was normalized to Montreal Neurologic Institute (MNI) space. The same diffeomorphism mapping was applied to the PET image, transforming it to MNI space. Both MRI and PET images were filtered to remove the background and skull using a brain mask in MNI space. SUVR map of ^18^F-AV45 PET was calculated relative to the cerebellar cortex. Moreover, values above the 99.7th percentile were capped (replace them with the 99.7th percentile value), and negative values were set to 0. Subsequently, the resolution of the MRI and PET images was converted to 256 × 256 × 256 by tiling and cropping.

### Deep learning

**Fig. 2:**
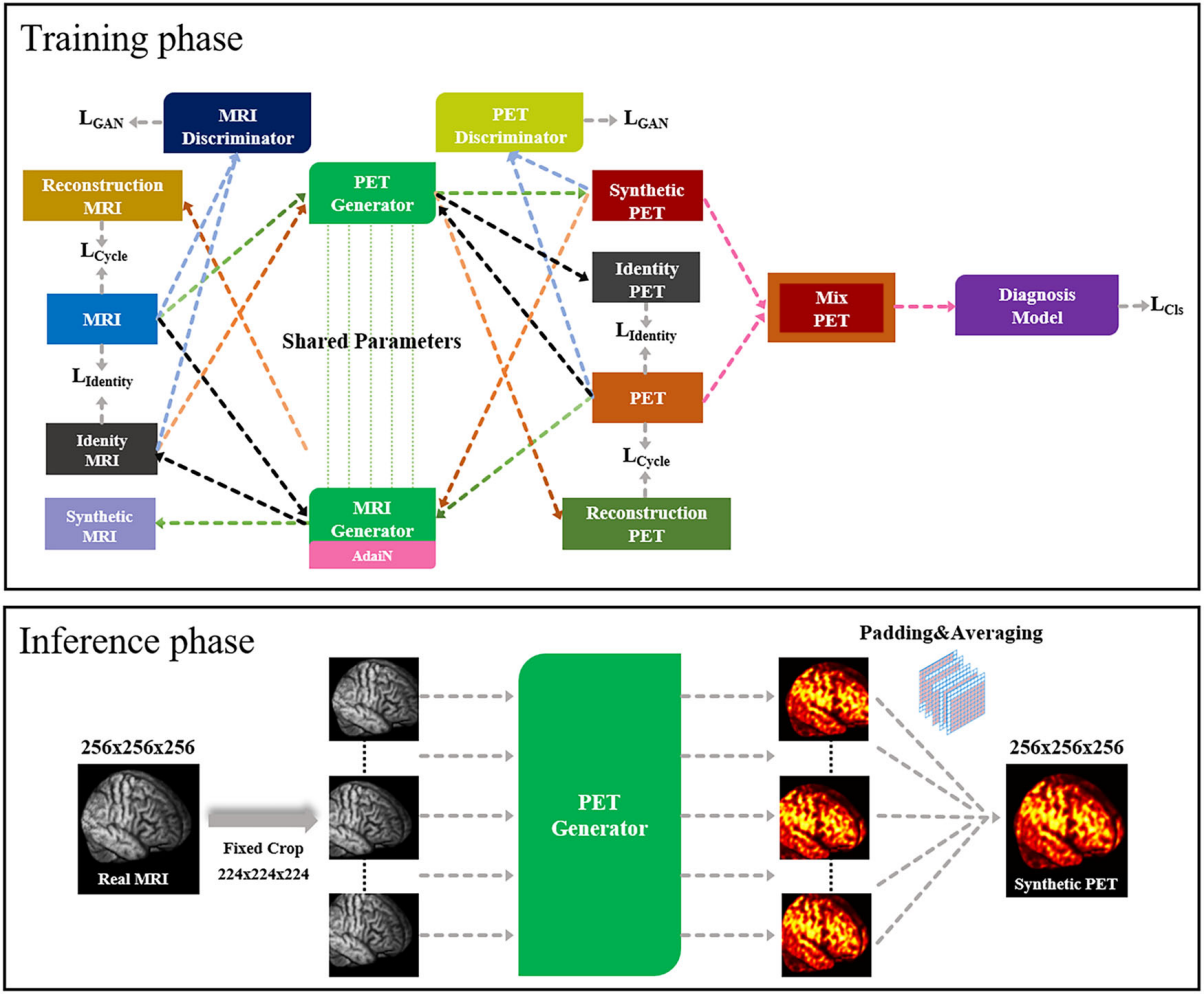
Deep learning method based on ShareGAN of 3D synthetic Aβ PET. Training phase illustrates the proposed framework of joint learning. Inference phase illustrates the logical process of synthetic PET from Real MRI using PET Generator. Synthetic PET and synthetic MRI: cross-modality images generated by feeding real data from one modality into the corresponding generator. Identity PET and identity MRI: Images reconstructed by feeding synthetic data back into the generator. Reconstruction PET and reconstruction MRI: Images reconstructed by feeding synthetic data back into the generator. MixPET: input of the Diagnosis model, composed of Synthetic/Identity PET and PET. Green arrows represent the workflow for cross-modal synthesis path. Orange arrows represent the workflow for reconstruction path. Black arrows represent the workflow for identity path. Blue arrows represent the workflow for discriminate path. Pink arrows represent the workflow for classifier path. L_GAN_, adversarial loss; L_Cycle_, cycle-consistency loss; L_identity_, identity loss; L_Cls_, classification loss; AdaIN, adaptive instance normalization; MRI, magnetic resonance imaging; Aβ, amyloid-β; PET, positron emission tomography; ShareGAN, generative adversarial network with share parameters

Our framework is a joint learning framework [27] of cross-modal synthesis (MRI to PET) and pretrained diagnosis network for radiation-free Aβ deposition assessment (Fig. 2). This 3D T1WI-based model, called GAN with share parameters (ShareGAN), learns the image contours, voxel intensities, and their spatial distributions to establish a mapping between structural MRI and corresponding Aβ PET images, enabling the generation of synthetic PET at anatomically matched locations.

Training occurs in two phases. Phase 1 (Pre-training): a 3D-DenseNet classifier [29] (input: 256 × 256 × 256) is trained on real PET for the classification of Aβ positives and negatives. Phase 2 (Joint Training): the frozen classifier co-optimizes a bidirectional synthesis network—using a single shared generator modulated by Adaptive Instance Normalization [30] to synthesize PET from MRI and MRI from PET (224 × 224 × 224 random crops). Synthetic PET and MRI are refined via adversarial loss (PatchGAN discriminators [31]). Besides, the synthetic PET is also refined via classification loss (3D-DenseNet classifier) for realism and diagnostic loss to align Aβ classification with real PET.

In the inference phase, input MRI (256 × 256 × 256) undergoes fixed-step cropping (224 × 224 × 224, stride 32). Sub-volumes are synthesized by the PET generator, then reconstructed into seamless full-brain PET (256 × 256 × 256) via mask-based padding and averaging overlapping regions. Further details of the model training and inference phase were provided in Supplementary Material Section 4.

### Model evaluation

The image quality of 3D synthetic Aβ PET was evaluated using quantitative metrics, including structural similarity index measure (SSIM), peak signal-to-noise ratio (PSNR), mean absolute error (MAE), and their respective averages were calculated.

In the external testing set 1 and external testing set 2, Pearson correlation coefficients and Bland–Altman analysis were performed for SUVR in meta-region of interest (ROI) between the synthetic and real Aβ PET images. Meta-ROI consisted of the frontal, temporal, parietal, precuneus, anterior striatum and insular cortex [32].

Both 3D synthetic Aβ PET and real Aβ PET were anonymized and randomly assigned to two senior radiologists who assessed the Aβ status and made an agreement, with a third party reviewing in case of disagreement. The Aβ status read from the real Aβ PET was considered as a ground truth.

### Longitudinal follow-up assessment

From Xuanwu Hospital data, 36 patients with AD/MCI underwent follow-up examinations 6 months after baseline PET/MR examinations. Using the baseline and follow-up data, we calculated the longitudinal change in SUVR for real PET and synthetic PET, respectively. Additionally, we conducted a correlation analysis to evaluate the relationship between the SUVR changes measured by the two imaging modalities.

### Statistical analysis

The differences between demographic characteristics were compared using SPSS 26 software. The Kolmogorov–Smirnov test was used to evaluate the distribution of continuous variables. To compare group differences in continuous variables, including SSIM, PSNR, and MAE between two groups, a two-sample *t*-test was used for data with a normal distribution, while the Wilcoxon test was applied for data that did not follow a normal distribution. One-way analysis of variance (ANOVA) was used to compare group differences in SSIM, PSNR, and MAE across more than two groups, with post hoc comparisons verifying the differences between any two groups. Statistical significance was determined at a level of *p* < 0.05.

The reliability of the Aβ deposition diagnosis was assessed using a confusion matrix. Accuracy, sensitivity, specificity, and F1 score were calculated to evaluate the diagnostic efficacy of the 3D synthetic Aβ PET.

## Results

### Characteristics of the datasets

The training set, validation set and external testing set 2 consisted of patients with AD, MCI and HC participants. And external testing set 1 consisted of patients with AD, MCI, HC participants, and 54 patients with other types of dementia, including VaD, bvFTD, DLB, and SD. Table 1 provided details of participant demographics.

**Table 1:** Participant demographics and clinical characteristics

Parameters	Training set	Validation set	External testing set 1	External testing set 2
No. of subjects	608	68	286	47
Age (years)	76.3 ± 7.5	76.3 ± 7.6	64.3 ± 8.1	66.7 ± 9.8
Gender (F/M)	330/278	29/39	179/107	29/18
Education (years)	16.5 ± 2.6	16.3 ± 2.7	11.6 ± 4.3	9.8 ± 4.6
MMSE score (n)	27.2 ± 3.0 (221)	26.7 ± 4.8 (27)	18.8 ± 7.3	21.7 ± 6.5 (42)
APOE ε4				
Carrier	232	26	65	3
Non-carrier	327	37	103	4
Aβ status				
Positive	294	33	177	33
Negative	314	35	109	14
Diagnosis category (n, Aβ positive)				
AD	46 (41)	5 (2)	144 (144)	25 (25)
MCI	246 (138)	30 (18)	64 (24)	14 (8)
HC	316 (115)	33 (13)	24 (0)	8 (0)
VaD	0	0	19 (3)	0
bvFTD	0	0	20 (0)	0
DLB	0	0	7 (6)	0
SD	0	0	8 (0)	0

Values are the total number or means ± standard deviation. The number in parentheses indicates the number of participants with that parameter

*F* female, *M* male, *MMSE* Mini-Mental State Examination, *APOE* apolipoprotein E, *Aβ* amyloid-β, *AD* Alzheimer’s disease, *MCI* mild cognitive impairment, *HC* healthy controls, *VaD* vascular dementia, *bvFTD* behavioral variant frontotemporal dementia, *DLB* dementia with Lewy bodies, *SD* semantic dementia

### Quantitative evaluation

**Fig. 3:**
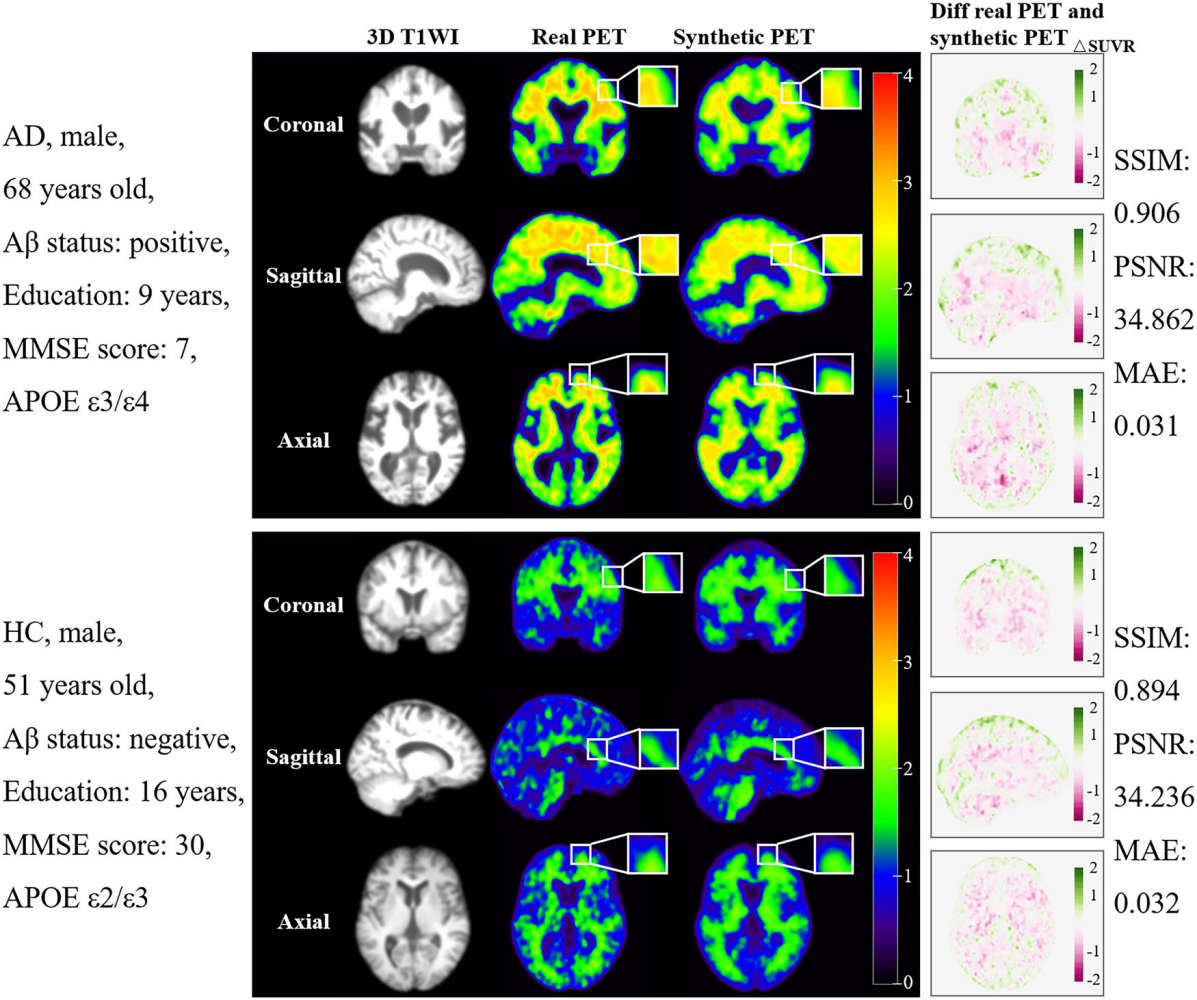
Representative model generated synthetic PET images for two subjects. In the panel 4 columns can be seen: the MRI input image (left), the real PET image (center-left), the synthetic PET image (center-right), and the error map between real and synthetic PET (right). The color bar of real and synthetic PET represents SUVR concentrations, and the error map color bar is the difference in SUVR. AD, Alzheimer’s disease; Aβ, amyloid-β; HC, healthy controls; MMSE, Mini-Mental State Examination; APOE, apolipoprotein E; 3D T1WI, high-resolution T1-weighted imaging; PET, positron emission tomography; diff, difference; △SUVR, the difference in standardized uptake value ratio; SSIM, structural similarity index measure; PSNR, peak signal-to-noise ratio; MAE, mean absolute error

Overall, a high degree of similarity between synthetic PET and real PET across the external testing set 1 was shown, with a mean SSIM = 0.898, PSNR = 34.690 dB and MAE = 0.031. In the quantitative evaluation of subgroups within the external testing set 1, the AD group exhibited SSIM = 0.895, PSNR = 34.473 dB, and MAE = 0.033; MCI exhibited SSIM = 0.900, PSNR = 34.854, and MAE = 0.030; and HC exhibited SSIM = 0.905, PSNR = 35.329, and MAE = 0.027. Additionally, the VaD group, bvFTD group, DLB group, and SD group showed high-quality performance across all indicators (Table 2). In external testing set 2, SSIM was 0.899, PSNR was 34.725 dB and MAE was 0.031. The observed SUVR values ranged from 0.836 to 2.130. Representative cases were shown in Fig. 3, with additional cases of various types of dementia available in Supplementary Material Section 5.

**Table 2:** Quantitative evaluation of 3D synthetic Aβ PET image quality

Groups	SSIM	PSNR	MAE
Validation set	0.898	34.696	0.031
External testing set 1	0.898	34.690	0.031
AD	0.895	34.473	0.033
MCI	0.900	34.854	0.030
HC	0.905	35.329	0.027
VaD	0.900	34.765	0.031
bvFTD	0.902	34.880	0.030
DLB	0.897	34.480	0.033
SD	0.901	34.913	0.030
External testing set 2	0.899	34.725	0.031

Higher values indicate better performance for SSIM and PSNR, whereas lower values indicate better performance for MAE

*Aβ* amyloid-β, *PET* positron emission tomography, *SSIM* structural similarity index measure, *PSNR* peak signal-to-noise ratio, *MAE* mean absolute error, *AD* Alzheimer’s disease, *MCI* mild cognitive impairment, *HC* healthy controls, *VaD* vascular dementia, *bvFTD* behavioral variant frontotemporal dementia, *DLB* dementia with Lewy bodies, *SD* semantic dementia

In addition, ANOVA showed that SSIM, PSNR, and MAE were significantly different among AD, MCI, and HC groups within external testing set 1 (*p* < 0.001, Table 3). SSIM and PSNR exhibited an increasing trend, while MAE exhibited a decreasing trend. This suggested that the image quality of synthetic PET in early AD was superior to that in late AD. Considering gender, there was a significant difference in SSIM (*p* = 0.005) and PSNR (*p* = 0.015) between males and females. Furthermore, SSIM was significantly different between male and female patients with AD (*p* = 0.026). No significant difference was found in MCI and HC. More details were given in Table 4. APOE ε4 had no significant effect on the AD and MCI groups (Supplementary Material Section 6).

**Table 3:** ANOVA-based comparison of 3D synthetic Aβ PET image quality across the AD, MCI, and HC groups

				*p*-value			
Parameters	AD	MCI	HC	ANOVA	AD vs. HC	AD vs. MCI	MCI vs. HC
SSIM	0.895 ± 0.007	0.900 ± 0.008	0.905 ± 0.006	< 0.001	< 0.001	< 0.001	0.005
PSNR	34.473 ± 0.561	34.854 ± 0.670	35.329 ± 0.563	< 0.001	< 0.001	< 0.001	0.001
MAE	0.033 ± 0.004	0.030 ± 0.004	0.027 ± 0.003	< 0.001	< 0.001	< 0.001	0.002

Values are means ± standard deviation except *p*-value

*Aβ* amyloid-β, *PET* positron emission tomography, *AD* Alzheimer’s disease, *MCI* mild cognitive impairment, *HC* healthy controls, *ANOVA* analysis of variance, *SSIM* structural similarity index measure, *PSNR* peak signal-to-noise ratio, *MAE* mean absolute error

**Table 4:** Two-sample *t*-test analysis of gender differences in 3D synthetic Aβ PET image quality across the AD, MCI and HC groups

Parameters	Male	Female	*p*-value
All participants			
SSIM	0.899 ± 0.007	0.896 ± 0.008	0.005**
PSNR	34.804 ± 0.640	34.587 ± 0.650	0.015*
MAE	0.031 ± 0.004	0.032 ± 0.004	0.080
AD group			
SSIM	0.897 ± 0.007	0.894 ± 0.007	0.026*
PSNR	34.574 ± 0.533	34.408 ± 0.562	0.094
MAE	0.033 ± 0.003	0.033 ± 0.004	0.248
MCI group			
SSIM	0.901 ± 0.008	0.899 ± 0.008	0.317
PSNR	34.923 ± 0.636	34.787 ± 0.684	0.270
MAE	0.0300 ± 0.0038^#^	0.0302 ± 0.0041^#^	0.837
HC group			
SSIM	0.905 ± 0.007	0.906 ± 0.004	0.503
PSNR	35.274 ± 0.655	35.421 ± 0.382	0.549
MAE	0.028 ± 0.004	0.027 ± 0.002	0.586

Values are means ± standard deviation except *p*-value; * *p* < 0.05; ** *p* < 0.01; ^#^ the results are presented to four decimal places to clearly demonstrate the differences between genders

*Aβ* amyloid-β, *PET* positron emission tomography, *AD* Alzheimer’s disease, *MCI* mild cognitive impairment, *HC* healthy controls, *SSIM* structural similarity index measure, *PSNR* peak signal-to-noise ratio, *MAE* mean absolute error

**Fig. 4:**
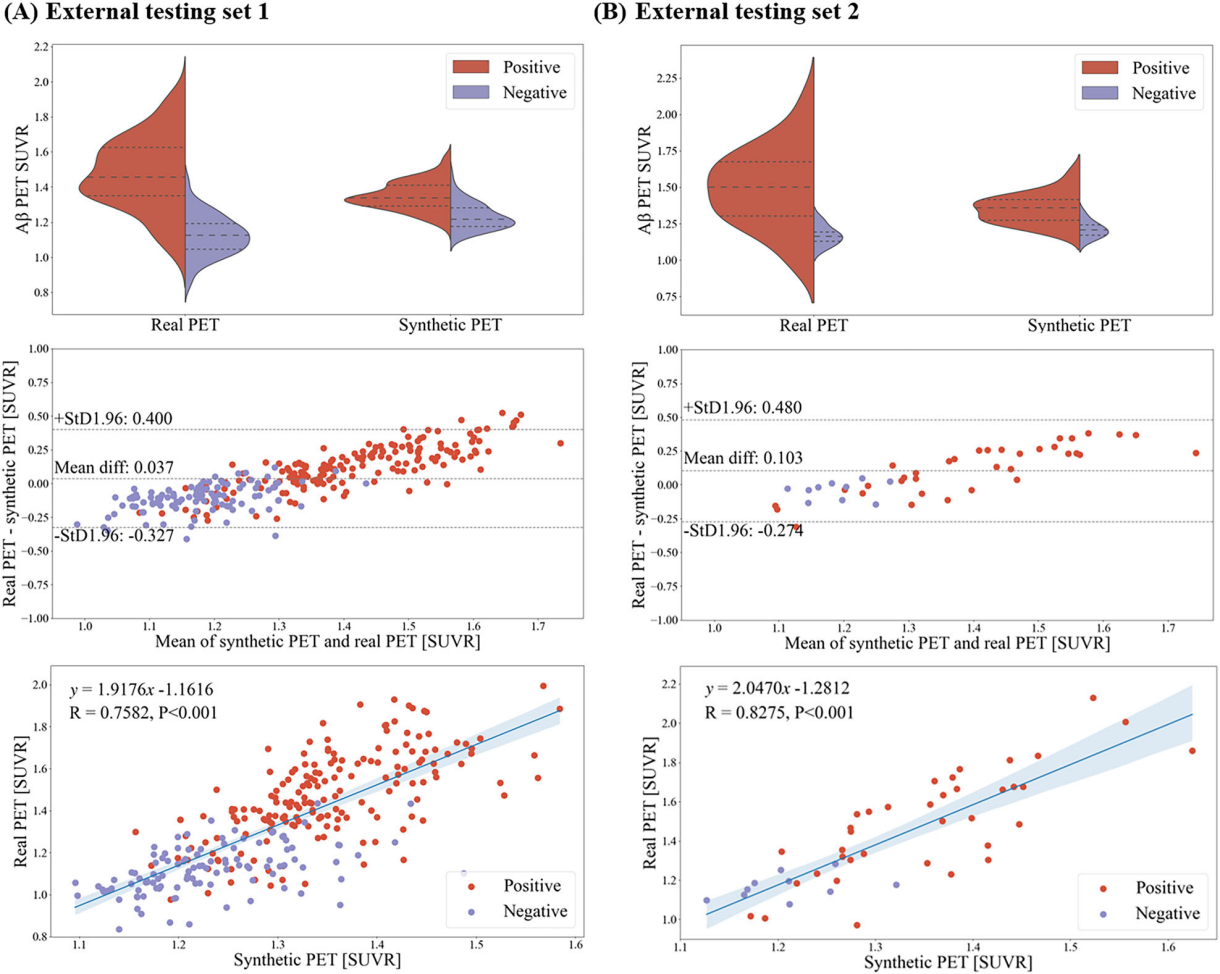
Assessment of 3D synthetic Aβ PET SUVR. Top, violin plots to show the distribution of Aβ PET SUVR both in synthetic and real PET. Middle, correlation plots with the linear regression equations, Pearson correlation coefficients (R), and *p*-value provided. Bottom, Bland–Altman plots to analyze any systematic differences between quantification by synthetic and real PET. **A** Violin plot, correlation plot, and Bland–Altman plot for external testing set 1. **B** Violin plot, correlation plot, and Bland–Altman plot for external testing set 2. Aβ, amyloid-β; PET, positron emission tomography; SUVR, standardized uptake value ratio; diff, difference; StD, standard deviation

Bland–Altman plots showed narrow limits of agreement (LoA) and no systematic bias (Fig. 4). For external testing set 1 (Fig. 4A), the mean difference (synthetic − real SUVR) was 0.037 (95% LoA: −0.327 to 0.400). For external testing set 2 (Fig. 4B), the mean difference was 0.103 (95% LoA: −0.274 to 0.480). While the mean differences were not statistically significant, the LoA width suggests moderate variability in agreement. 3D synthetic Aβ PET showed significant correlation with real Aβ PET in SUVR for quantifying Aβ deposition in the external testing set 1 (R = 0.758, *p* < 0.001, Fig. 4A) and external testing set 2 (R = 0.828, *p* < 0.001, Fig. 4B). Moreover, voxel-wise Pearson correlation analysis across the whole brain revealed a mean correlation coefficient of R = 0.771 (*p* < 0.001) in external testing set 1 and R = 0.785 (*p* < 0.001) in external testing set 2 (Supplementary Material Section 7).

### Diagnostic evaluation

A total of 286 3D synthetic Aβ PET images from subjects at Xuanwu Hospital were used for clinical visual diagnosis to assess their diagnostic performance. In the external testing set 1, the overall accuracy of the synthetic images was 88.5%, with a sensitivity of 97.2%, specificity of 74.3%, and F1 score of 91.2%. The accuracy for the subgroups was as follows: 96.5% for the AD group, 84.4% for the MCI group, 75% for the HC group, 73.7% for the VaD group, 75.0% for the bvFTD group, 100% for the DLB group, and 75.0% for the SD group. More details of diagnosis based on synthetic PET, including the confusion matrix, were presented in Table 5 and Supplementary Material Section 8.

**Table 5:** Diagnostic performance of 3D synthetic Aβ PET for assessing Aβ status across dementia types

Parameters	AD	MCI	HC	VaD	bvFTD	DLB	SD
Accuracy	96.5	84.4	75.0	73.7	75.0	100	75.0
Sensitivity	96.5	100	/	100	/	100	/
Specificity	/	73.2	75.0	68.8	75.0	100	75.0
F1 score	/	81.4	/	54.5	/	100	/

Values are presented in percent; “/” means none of this parameter

*Aβ* amyloid-β, *PET* positron emission tomography, *AD* Alzheimer’s disease, *MCI* mild cognitive impairment, *HC* healthy controls, *VaD* vascular dementia, *bvFTD* behavioral variant frontotemporal dementia, *DLB* dementia with Lewy bodies, *SD* semantic dementia

Synthetic PET and real PET images from 47 subjects at Nanjing First Hospital and the First Hospital of Hebei Medical University were used as the external testing set 2 to evaluate diagnostic performance. The accuracy was 89.4%, the sensitivity was 93.9%, the specificity was 78.6%, and the F1 score was 92.5%.

### Longitudinal changes evaluation

**Fig. 5:**
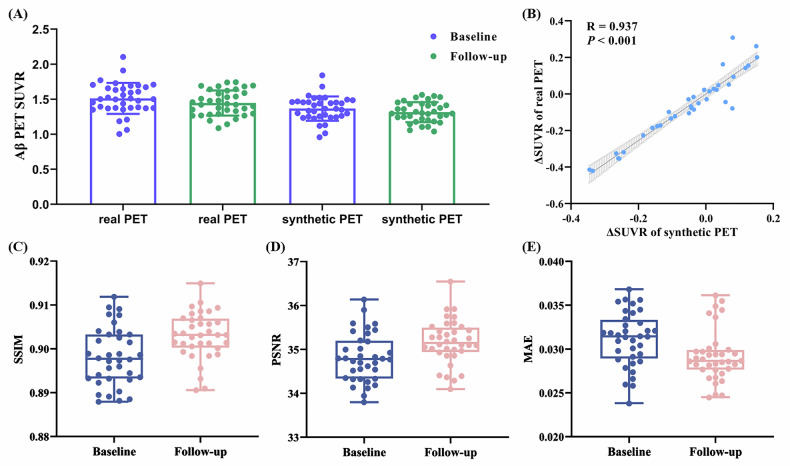
Analysis for follow-up data of synthetic Aβ PET. **A** Distribution of Aβ PET SUVR at baseline and 6-month follow-up. **B** Correlation between SUVR changes measured by synthetic PET and those measured by real PET. Distribution of SSIM (**C**), PSNR (**D**), and MAE (**E**) for synthetic PET images acquired at baseline and 6 months follow-up. Aβ, amyloid-β; PET, positron emission tomography; SSIM, structural similarity index measure; PSNR, peak signal-to-noise ratio; MAE, mean absolute error; SUVR, standardized uptake value ratio; △, difference

The follow-up analysis of real Aβ PET showed a mean SUVR change of −0.065, compared to −0.052 for synthetic Aβ PET. The correlation of longitudinal SUVR changes between synthetic PET and real PET was R = 0.937, *p* < 0.001. The synthetic PET at follow-up had SSIM = 0.903, PSNR = 35.172 dB, and MAE = 0.029 (Fig. 5).

## Discussion

In this work, we developed a ShareGAN-based image-to-image model for brain PET that generates 3D Aβ PET-like images from 3D T1WI, providing a non-invasive, accessible, and cost-effective complementary approach to real Aβ PET imaging. In the quantitative image quality evaluation, the 3D synthetic Aβ PET images generated by the ShareGAN model were comparable to real Aβ PET images. Diagnosis based on synthetic PET offered good and stable diagnostic accuracy for Aβ status in AD, as well as in other types of dementia, including VaD, bvFTD, DLB, and SD. For the analysis of Aβ PET SUVR, synthetic PET showed high concordance with real PET and yielded a strong correlation with longitudinal changes in follow-up data. These findings indicate that the proposed model may serve as a supportive tool in AD diagnosis and monitoring, helping to identify patients who require further Aβ PET imaging.

In terms of quantitative image assessment metrics, including SSIM, PSNR, and MAE, our synthetic PET had a high degree of reproduction for Aβ PET. SSIM reflects the overall performance of an image in terms of structure, luminance, and contrast, while PSNR is the most widely used evaluation metric based on differences in image pixels [33]. In comparison to previous synthetic Aβ PET studies (SSIM = 0.905, PSNR = 22.685) [34], our study had similar SSIM (0.898) and significant improvement in PSNR (34.690). Importantly, the synthetic Aβ PET in their study was a 2D image trained within a 2D space using axial plane images. In contrast, our approach generated 3D synthetic PET that closely resembles clinical 3D PET, thereby preserving critical information across all planes. This highlights our study’s emphasis on enabling comprehensive 3D analysis, which ensures precise results. Ou et al [35] similarly generated Aβ PET from 3D T1WI, using a dataset comprising 82 patients with AD, 125 patients with MCI, and 303 HC participants. Compared to their model, our model achieved a 33.5% improvement in PSNR, while maintaining comparable performance in SSIM and MAE. Regarding diagnostic accuracy for Aβ status, their model reached an accuracy of 86.6% versus our 88.5%, indicating comparable diagnostic effectiveness. Importantly, our study went a step further by conducting subgroup analysis in patients with MCI, demonstrating that our model also holds promise for application in the early stages of AD. Furthermore, evaluation across additional dementia subtypes offers preliminary support for the model’s potential generalizability to diverse dementia populations.

Bland–Altman analysis demonstrated good agreement between synthetic and real PET SUVR values, with relatively narrow limits of agreement. The positive slope observed in the plots may be attributed to the intensity normalization step in the model, which capped SUVR values at the 99.7th percentile to stabilize training and reduce the influence of extreme outliers, thereby enhancing model robustness. In addition, we observed that the SUVR distribution in synthetic PET was more compact than that of real PET, which is likely due to the same reason. However, this did not affect the visual interpretability of the images, and the overall diagnostic performance of the synthetic PET remained high, supporting its suitability for Aβ assessment in AD. It is worth noting that the diagnostic specificity was 73.2% for the MCI group and 75.0% for the HC group, indicating a potential risk of false-positive Aβ classification in these populations. However, as the proposed method is intended to serve as a supportive tool rather than a replacement for real PET, it may allow 73.2% of individuals in the MCI group and 75.0% in the HC group to avoid unnecessary PET-related radiation exposure. The remaining 26.8% and 25.0%, respectively, could undergo confirmatory PET scans to mitigate the risk of false positives. Our study demonstrated an overall accuracy of 88.5% on the external testing set 1 and 89.3% on the external testing set 2. Accuracy for the AD, MCI, HC, VaD, bvFTD, DLB, and SD groups ranged from 73.7 to 100%. Moreover, the good diagnostic performance of our model across various types of dementia suggests that, with training on larger sample sizes, our synthetic Aβ PET model holds potential for supplementing Aβ PET data in dementia patients who lack access to such imaging. The synthetic PET developed in this study allowed to provide a distribution of Aβ deposition that highly resembles real Aβ PET. This provides reliability and interpretability for visual diagnosis and further quantitative analysis.

The synthetic PET generated in this study also supports quantitative analysis. In terms of the Aβ PET SUVR parameter, synthetic PET exhibited high consistency and strong correlation with real PET in both external testing sets 1 and 2. Additionally, our longitudinal follow-up data showed that changes in SUVR measured by synthetic PET over time were highly correlated with those measured by real PET. All patients in the follow-up group received lecanemab treatment, and the mean SUVR values decreased at the 6-month follow-up compared to baseline, as measured by both real and synthetic PET. These findings suggest that synthetic PET may serve as a complementary tool for monitoring treatment effects, while potentially reducing patients’ exposure to PET-related radiation.

Gender, as a critical factor in AD, also significantly influenced our ShareGAN model. In the AD group, we found that the SSIM and PSNR of male subjects (SSIM = 0.899; PSNR = 34.804 dB) were higher than those of female subjects (SSIM = 0.896, *p* = 0.005; PSNR = 34.587 dB, *p* = 0.015). Further analysis among patients with AD, MCI, and HC participants revealed that male patients with AD (SSIM = 0.897) had a higher SSIM than female patients with AD (SSIM = 0.894, *p* = 0.026). Past studies have indicated that women face a higher risk and severity of AD [36–38]. This suggests that the difference in image quality may be due to more severe brain atrophy caused by AD in female patients. This also explains the results of our study that SSIM and PSNR showed a decreasing trend with the progression of disease in HC, MCI, and AD (*p* < 0.001). No gender differences in image quality were noted in MCI and HC, possibly due to their earlier disease stages and smaller participant numbers compared to AD. Nonetheless, we emphasize that these statistical variations are unlikely to impact clinical utility or human visual interpretation of the generated images.

This study has several limitations. First, the number of patients with VaD, bvFTD, DLB, and SD only occupied a small proportion of the recruited subjects. Therefore, the generality of the ShareGAN-based image-to-image model in this study in non-AD dementia requires a larger sample size for verification. Second, although our model demonstrated the ability to track longitudinal Aβ changes, the longitudinal evaluation was based on a modest sample size (*n* = 36), and further studies with larger follow-up cohorts are warranted to confirm the robustness of temporal performance. Finally, as the proposed method is intended to serve as a supportive tool rather than a replacement for real PET, the diagnostic specificity of the synthetic PET for identifying Aβ status in MCI requires further improvement through increased sample size and algorithm optimization.

In conclusion, we constructed a ShareGAN-based image-to-image model for generating 3D synthetic Aβ PET from 3D T1WI, demonstrating comparable image quality to real PET and achieving satisfactory diagnostic accuracy for Aβ status. With further refinement and validation, this model has the potential to become a promising, rapid and cost-effective auxiliary tool for conducting Aβ PET-like imaging in clinical settings. It could help identify, among high-risk individuals with subjective or suspected cognitive impairment who are candidates for PET, those who are truly in need of real Aβ PET scans, thereby improving the accessibility of early AD screening.

## Supplementary information

ELECTRONIC SUPPLEMENTARY MATERIAL

## Abbreviations

^18^F-AV45: ^18^F-florbetapir
^18^F-FDG: ^18^F-fluorodeoxyglucose
3D T1WI: High-resolution T1-weighted imaging
AD: Alzheimer’s disease
ADNI: Alzheimer’s Disease Neuroimaging Initiative
APOE: Apolipoprotein E
Aβ: Amyloid-β
bvFTD: Behavioral variant frontotemporal dementia
CSF: Cerebrospinal fluid
DLB: Dementia with Lewy bodies
GAN: Generative adversarial network
HC: Healthy controls
MAE: Mean absolute error
MCI: Mild cognitive impairment
MNI: Montreal Neurologic Institute
PSNR: Peak signal-to-noise ratio
ROI: Region of interest
SD: Semantic dementia
ShareGAN: Generative adversarial network with share parameters
SSIM: Structural similarity index measure
SUVR: Standardized uptake value ratio
VaD: Vascular dementia
