# Supplementary material for: MRI-to-PET synthesis via deep learning for amyloid-β quantification in Alzheimer’s disease
Source: Eur Radiol. 2026 Jan 7;36(6):5125–37. doi: 10.1007/s00330-025-12251-3 (PMC13212758; doi:10.1007/s00330-025-12251-3)
Supplement: Supplementary file 1 — ELECTRONIC SUPPLEMENTARY MATERIAL [file 330_2025_12251_MOESM1_ESM.pdf]

MRI-to-PET Synthesis via Deep Learning for Amyloid-β Quantification in Alzheimer's Disease

ELECTRONIC SUPPLEMENTARY MATERIAL

Outline

1 Inclusion criteria

1.1 Alzheimer’s Disease Neuroimaging Initiative

1.2 Hospitals data

2 Exclusion criteria

3 Acquisition parameters

3.1 Xuanwu Hospital, Capital Medical University

3.2 Nanjing First Hospital, Nanjing Medical University

3.3 The First Hospital of Hebei Medical University

4 Method of deep learning

4.1 Joint learning model input and output

4.1.1 Diagnosis model and pre-training

4.1.2 Synthesis model and joint learning

5 Representative cases

6 Quantitative Evaluation

7 Diagnostic Evaluation

Supplement material references

1 Inclusion criteria

(1) Magnetic resonance imaging (MRI): 3D scan, slice thickness < 1.65mm; (2) MRI: T1-weighted; (3) Field strength: 1.5~3.0T; (4) Positron emission tomography (PET): paired with MRI for same person, and time span between MRI and PET scans was less than 30 days; (5) PET: radiotracer was ^{18}F-florbetapir (^{18}F-AV45); (6) PET: Slice thickness < 5mm; (7) The following diagnostic criteria were met (**1.1~1.2.5**).

1.1 Alzheimer's Disease Neuroimaging Initiative

Participants included in this study ranged from health control (HC) to those with MCI and Alzheimer's disease (AD). The diagnostic criteria for participants from Alzheimer's Disease Neuroimaging Initiative (ADNI) can be found at https://adni.loni.usc.edu/wpcontent/uplo-ads/2010/09/ADNI_GeneralPro-ceduresManual.pdf.

1.2 Hospitals data

1.2.1 Alzheimer's disease

For participants from hospitals, the diagnosis of AD was based on the National Institute of Neurological and Communicative Diseases and Stroke/Alzheimer's Disease and Related Disorders Association criteria for probable[1]. The diagnosis of MCI was based on diagnostic criteria defined by Petersen et al[2]. The HC participants were age- and gender-matched to patients and had no cognitive decline complaints, depression, or anxiety, with the Mini-Mental State Examination (MMSE) score ≥ 26.

1.2.2 Vascular dementia

Participants diagnosed with vascular dementia (VaD) met the criteria of the National Institute of Neurological Disorders and Stroke-Association Internationale pour la Recherche et l’Enseignement en Neurosciences[3].

1.2.3 Behavioural variant frontotemporal lobe dementia

Participants diagnosed with behavioural variant frontotemporal lobe dementia (bvFTD) met the diagnostic criteria revised by Rascovsky et al.[4] based on the International Behavioural Variant FTD Criteria Consortium (FTDC).

1.2.4 Dementia with Lewy bodies

Participants diagnosed with dementia with Lewy bodies (DLB) met the most recent revised diagnosis criteria for probable DLB, established by McKeith et al[5].

1.2.5 Semantic dementia

Participants diagnosed with semantic dementia (SD) met the criteria proposed by Gorno-Tempini et al.[6] in 2011.

2 Exclusion criteria

(1) MRI and PET image files were not in Digital Imaging and Communications in Medicine (DICOM) format; (2) There was some confusing information about the radiotracer; (3) Poor image quality scans, including those with head motion artifacts.

3 Acquisition parameters

3.1 Xuanwu Hospital, Capital Medical University

3.1.1 PET/MR 3.0-Tesla system (uPMR 790, United Imaging Healthcare)

The high-resolution T1-weighted imaging (3D T1WI) with following parameters: repetition time (TR) of 7.2 ms, echo time (TE) of 3.0 ms, flip angles (FA) of 10°, and voxel size of 1.0 × 1.0 × 1.0 mm^3. The PET data used ^{18}F-AV45 as tracer with matrix size of 192 × 192, field view of 250 mm, voxel size of 1.3 × 1.3 × 2.8 mm^3.

3.1.2 PET/MR 3.0-Tesla system (Signa, GE Healthcare)

The 3D T1WI with following parameters: TR of 7.0 ms/8.5 ms, TE of 3.0 ms/3.2 ms, FA of 12°/15°, and voxel size of 1.0 × 1.0 × 1.0 mm^3. The PET data used ^{18}F-AV45 as tracer with matrix size of 192 × 192, field view of 350 mm, voxel size of 1.8 × 1.8 × 2.8 mm^3.

3.2 Nanjing First Hospital, Nanjing Medical University

3.2.1 PET/MR 3.0-Tesla system (uPMR 790, United Imaging Healthcare)

The 3D T1WI with following parameters: TR of 7.2 ms, TE of 3.0 ms, FA of 10°, and voxel size of 0.7 × 0.7 × 0.7 mm^3. The PET data used ^{18}F-AV45 as tracer with matrix size of 256 × 256, field view of 300 mm, voxel size of 1.2 × 1.2 × 1.4 mm^3.

3.3 The First Hospital of Hebei Medical University

3.3.1 Siemens Magnetom Prisma 3T scanner

The 3D T1WI with following parameters: TR of 1800 ms, TE of 2.3 ms/2.2 ms, FA of 8°, and voxel size of 1.0 × 1.0 × 1.0 mm^3/1.0 × 1.0 × 1.3 mm^3.

3.3.2 Siemens Biograph mCT PET scanner

The PET data used ^{18}F-AV45 as tracer with matrix size of 400 × 400, field view of 400 mm, voxel size of 1.0 × 1.0 × 1.0 mm^3/1.0 × 1.0 × 3.0 mm^3.

4 Method of deep learning

4.1 Joint learning model input and output

The image after data preprocessing used for model training was shown in Fig S1. As shown in Fig S2, the input size of diagnosis network was 256 × 256 × 256, and the input/output size for the synthesis network was 224 × 224 × 224, since we used random cropping from 256 × 256 × 256 to 224 × 224 × 224 for data augmentation and to avoid the problem of insufficient GPU memory. During the joint learning phase, the synthesized outputs were padding to 256 × 256 × 256 based on the corresponding crop mask, and used the corresponding value from cropped PET to replace constant padding operation.

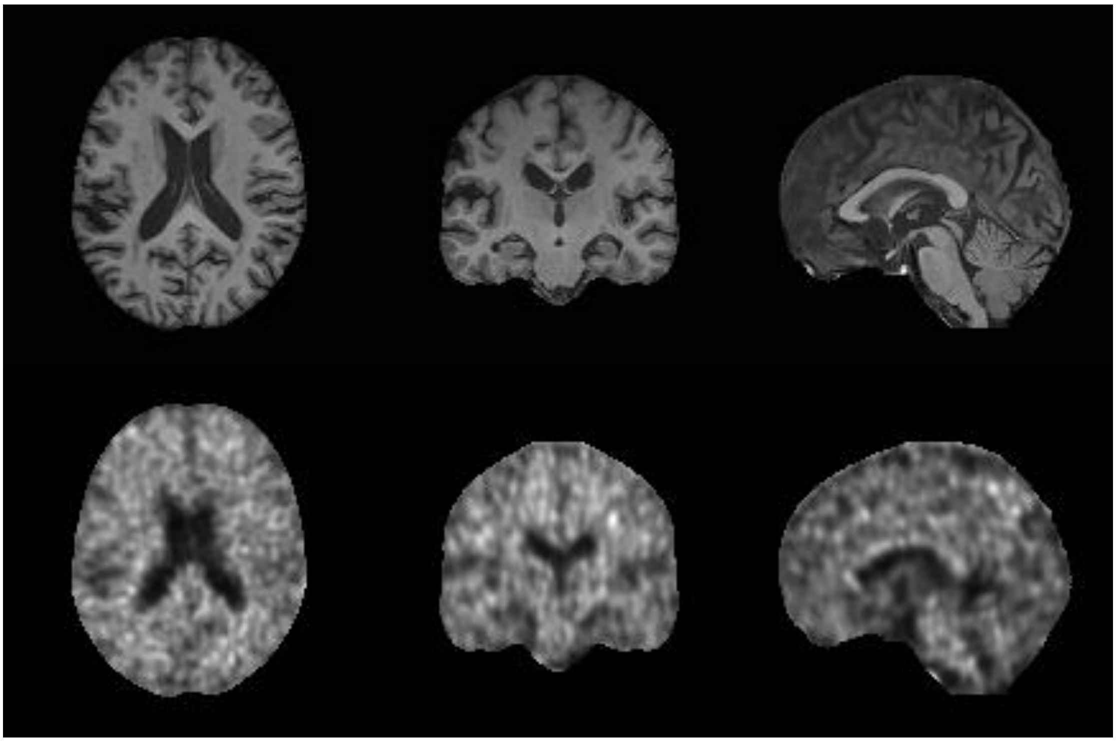

Fig S1: The final preprocessed MRI (up) and PET (down) on axial (left), coronal (middle), and sagittal (right) views.

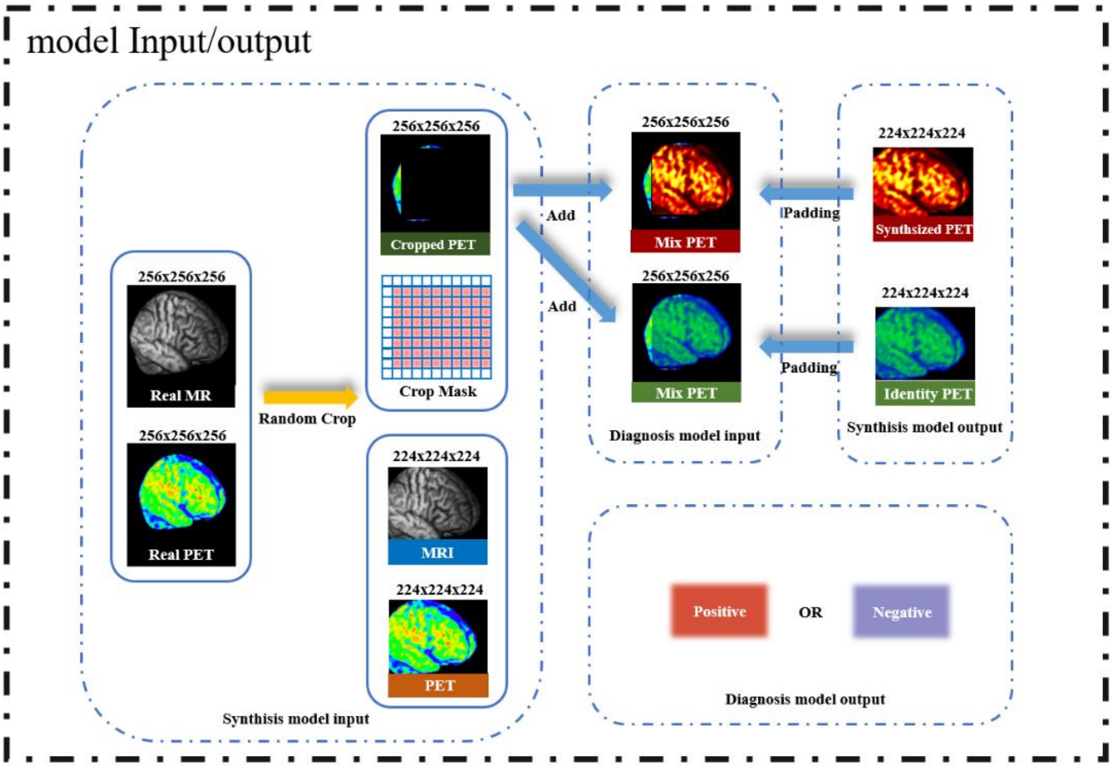

Fig S2: Illustration of the model input and output.

4.1.1 Diagnosis model and pre-training

As shown in Fig S3, the 3D-DenseNet consisted of a Conv3d Block (kernel size = 7, stride = 2, padding = 3), 3d max pooling layer (kernel size = 3, stride = 2, padding =1), followed by 4 dense blocks and 3 transition blocks. The 4 dense blocks consisted of 3, 6, 12 and 8 dense layers, respectively (denoted above each block). The growth rate (k) in the dense blocks was 12. The compression factor (θ) in the transition blocks is 0.6.

The CrossEntropy Loss was used as the loss function. The model was optimized using the Adam optimizer with the parameters: $\beta 1 = 0.5$ and $\beta 2 = 0.999$. The kaiming initialization strategy was used for weight initialization. The max training epoch was 500, using the early stopping method (patience = 10, delta = 0.01) to select better network weights by the validation set. The learning rate was 1×10^{-4} and decreased by a factor of 2 for every 10 epochs, and the min learning rate=1×10^{-6}.

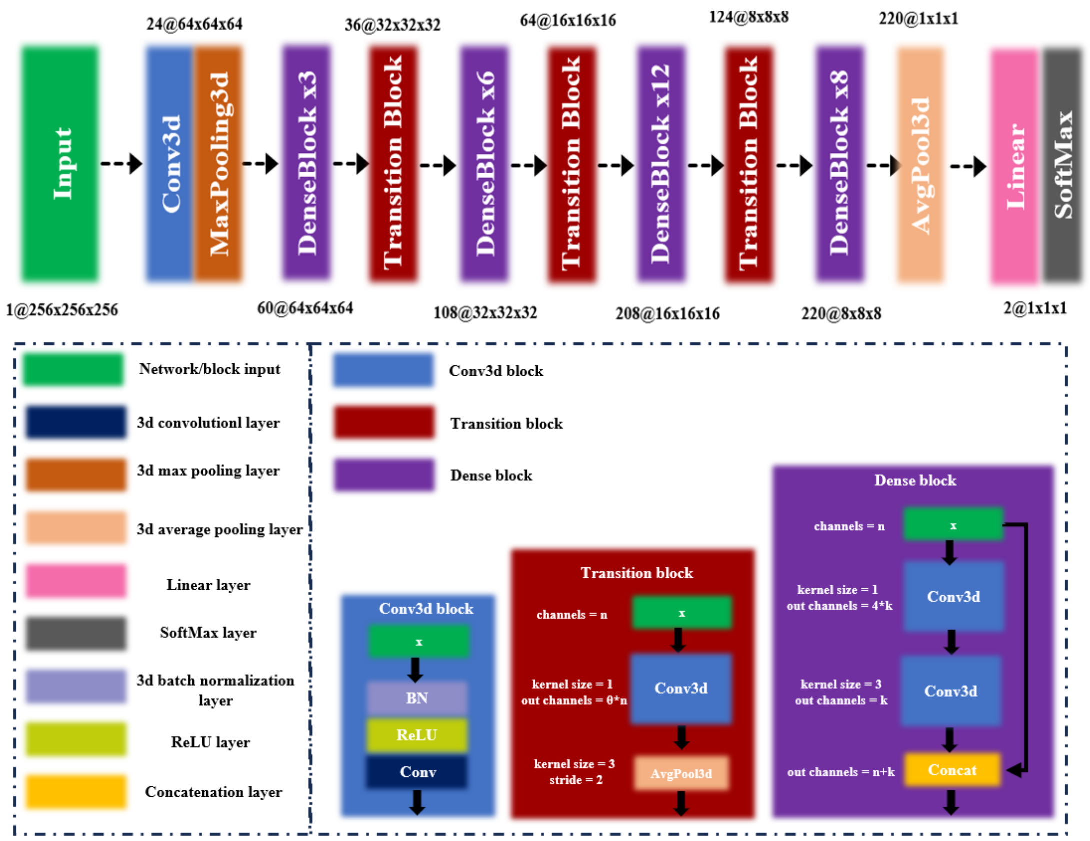

Fig S3: Network structure of 3D-DenseNet.

4.1.2 Synthesis model and joint learning

The generator structure consisted of a Adaptive Instance Normalization (AdaiN) module, seven Conv3d blocks and AdaiN branch blocks, a self-attention block, four transposed convolutional layers, a 3d convolutional layer and Tanh layer (Fig S4A). For the Conv3d block, kernel size = 3 and padding = 1, and the stride = 2 if down-sampling, else stride = 1. The kernel size = 4, stride = 2 and padding = 1 for both of transposed convolutional layers. In the AdaiN branch block, parameter α was used to specify the conversion direction of the generate function, α = 1 when the generator was used to synthesis MRI from PET, and α = 0 when the generator was used to synthesis PET from MRI. The

number of heads = 8 in the self-attention block, and the self-attention operation can be decomposed into two steps with a fully convolution-implemented[7].

Six Conv3d blocks and Leaky ReLU layers was used in the discriminator, and a 3d convolutional layer was used as the last layer to generate the final feature map of size 7 × 7 × 7 (Fig S4B). Both of the convolution operation using kernel size = 3, padding =1, and the stride = 2 if down-sampling, else using stride = 1.

The Objective function was combined with three different losses, including adversarial loss (L_{GAN}), cycle-consistency loss (L_{Cycle}), identity loss (L_{Cycle}) and classification loss (L_{Cls}):

$$L = L_{GAN} + \lambda_{Cycle*}L_{Cycle} + \lambda_{Ide*}L_{Ide} + \lambda_{Cls*}L_{Cls}$$

where λCycle, λIde and λCls are hyperparameters that adjust the weights between these three objective functions, and set to 5, 0.5 and 1, respectively, in our experiments.

Further, LSGAN loss[8] was used as adversarial loss for two modality discriminators Dm (MRI Discriminator) and Dp (PET Discriminator) to distinguish the authenticity of synthesized and real images.L1 loss was used as Cycle-consistency loss and Identity loss to constrains the conversion between original and transformed modalities, and forces the generate model Gm (MRI Generator) and Gp (PET Generator) to achieve an identity mapping from the input to the output such as Gp (xp) ≈ xp, where xp means the real PET input image. The CrossEntropy Loss was used as the classification loss.

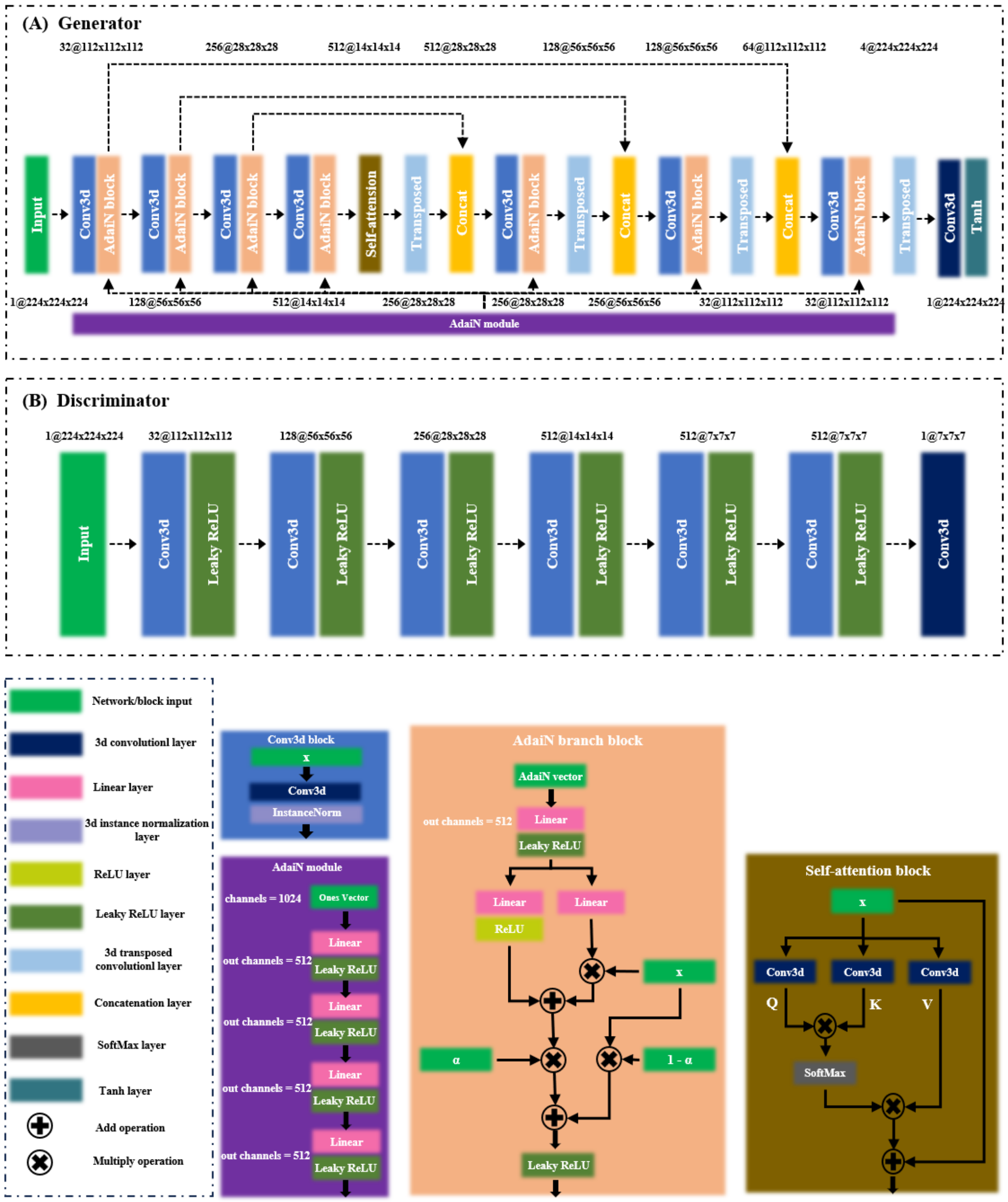

Fig S4: Network structure of generator and discriminator.

The learning rate for the diagnosis model was set to zero, since it only used to provide the classification loss for optimizing the synthesis model. The synthesis model and discriminators were optimized using the Adam optimizer with the parameters: β1 = 0:5 and β2 = 0.999. The learning rate was 1 × 10^{-4}

and decreased by a factor of 2 for every 50 epochs, and the min learning rate=1 × 10^{-6}. The kaiming initialization strategy was used for weight initialization. The training epoch was 1000, using the early stopping method (patience = 10, delta = 0.01) to select better network weights by the validation set. The neural network was implemented using Pytorch library. Training and testing were performed on two GeForce RTX 3090 Ti GPU.

5 Representative cases

Representative cases of AD and VaD were shown in Fig S5. Representative cases of bvFTD, DLB, and SD were shown in Fig S6.

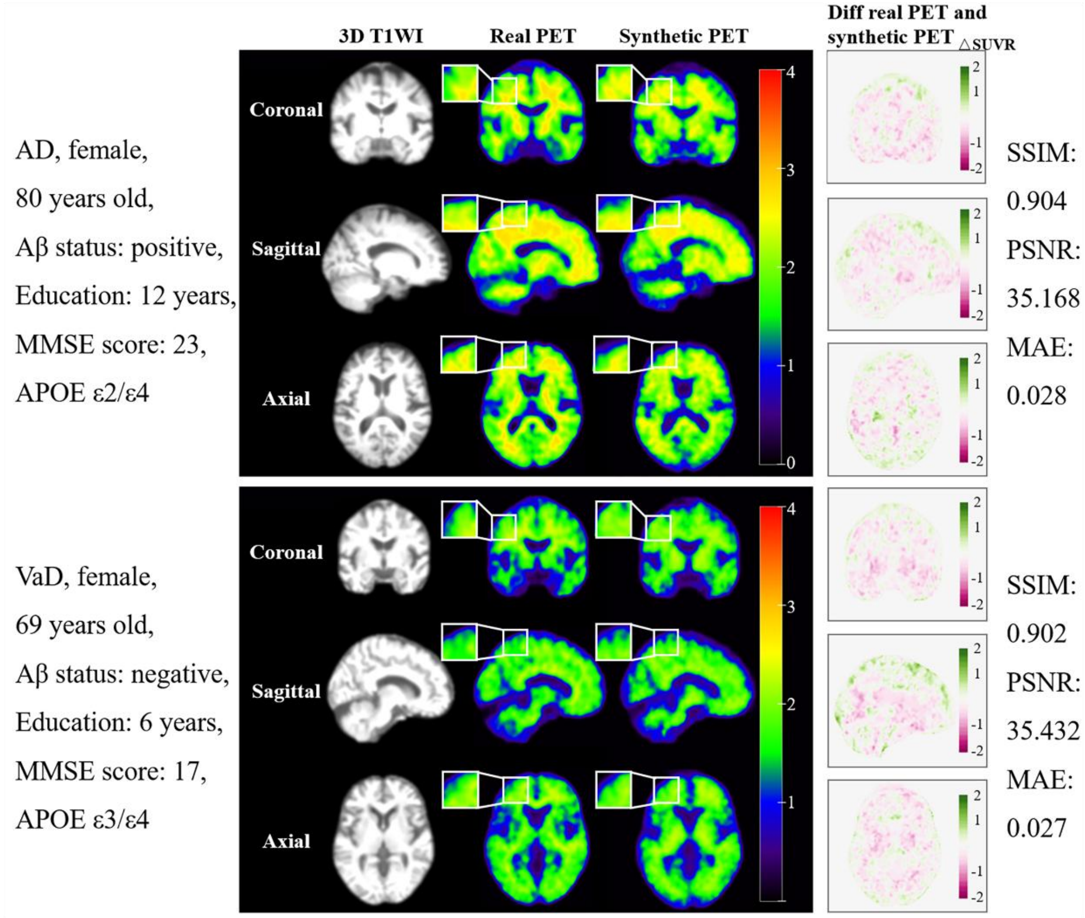

Fig S5: Representative model generated synthetic PET images for two subjects. Each panel shows four columns: the MRI input image (left), the real PET image (center-left), the synthetic PET image (center-right), and the error map between real and synthetic PET (right). The color bar of real and synthetic PET represents SUVR concentrations, and the error map color bar is the difference in SUVR. AD, Alzheimer's disease; VaD, vascular dementia; Aβ, amyloid-β; MMSE, Mini-Mental State Examination; APOE, apolipoprotein E; 3D T1WI, high-resolution T1-weighted imaging; PET, positron emission tomography; diff, difference; △SUVR, the difference in standardized uptake value ratio; SSIM, structural similarity index measure; PSNR, peak signal-to-noise ratio; MAE, mean absolute error.

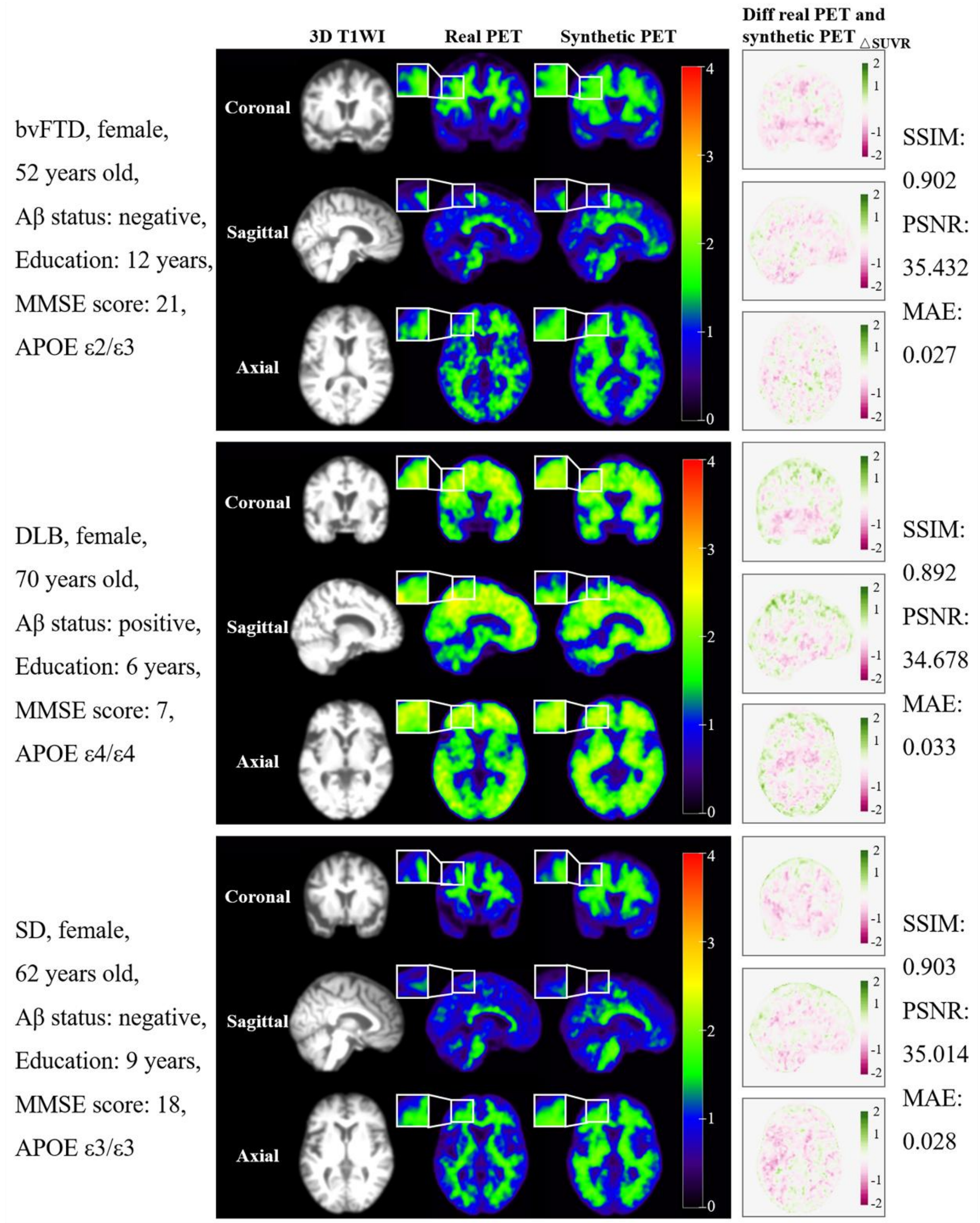

Fig S6: Representative model generated synthetic PET images for three subjects. Each panel shows four columns: the MRI input image (left), the real PET image (center-left), the synthetic PET image (center-right), and the error map between real and synthetic PET (right). The color bar of real and synthetic PET represents SUVR concentrations, and the error map color bar is the difference in SUVR. bvFTD, behavioural variant frontotemporal dementia; DLB, dementia with Lewy bodies; SD, semantic dementia; Aβ, amyloid-β; MMSE, Mini-Mental State Examination; APOE, apolipoprotein E; 3D T1WI, high-resolution T1-weighted imaging; PET, positron emission tomography; diff, difference; △SUVR, the difference in standardized uptake value ratio; SSIM, structural similarity index measure; PSNR, peak signal-to-noise ratio; MAE, mean absolute error.

6 Quantitative Evaluation

Apolipoprotein E (APOE) ε4 was considered the most critical risk gene in AD. For AD and MCI group, there was no significant difference in term of Structural Similarity Index Measure (SSIM), Peak Signal-to-Noise Ratio (PSNR), and Mean Absolute Error (MAE) between APOE ε4 carriers and non-carriers (Table S1). No group differences were calculated for SSIM, PSNR, and MAE because there was only one APOE ε4 carrier in the HC group.

Table S1: APOE differences in 3D synthetic Aβ PET images quality within AD, MCI, and HC groups

Parameters	APOE ε4 carrier	APOE ε4 non-carrier	*P* value
All participants			
SSIM	0.8959 ± 0.0075[#]	0.8958 ± 0.0073[#]	0.970
PSNR	34.545 ± 0.639	34.525 ± 0.639	0.876
MAE	0.033 ± 0.004	0.032 ± 0.004	0.799
AD group			
SSIM	0.896 ± 0.007	0.893 ± 0.006	0.078
PSNR	34.520 ± 0.589	34.296 ± 0.520	0.064
MAE	0.033 ± 0.004	0.034 ± 0.004	0.154
MCI group			
SSIM	0.895 ± 0.008	0.899 ± 0.006	0.082
PSNR	34.533 ± 0.725	34.889 ± 0.566	0.125
MAE	0.032 ± 0.004	0.030 ± 0.003	0.083
HC group			
SSIM	0.914	0.904 ± 0.008	/
PSNR	35.761	35.133 ± 0.805	/
MAE	0.024	0.028 ± 0.005	/

Note: Values are means ± standard deviation except the values of APOE ε4 carrier in HC group and *P* value; [#], the results are presented to four decimal places to clearly demonstrate the differences between APOE carriers and non-carriers; /, means none.

Abbreviation: APOE, apolipoprotein E; Aβ, amyloid-β; PET, positron emission tomography; SSIM, structural similarity index measure; PSNR, peak signal-to-noise ratio; MAE, mean absolute error; AD, Alzheimer's disease; MCI, mild cognitive impairment; HC, health control.

7 Voxel-wise Correlation Analysis

For each subject, a voxel-wise Pearson correlation analysis was performed between synthetic and real PET images. The results showed that the mean correlation coefficient was R = 0.771 ± 0.067 (all p < 0.001) in external testing set 1 and R = 0.785 ± 0.067 (all p < 0.001) in external testing set 2 (Fig. S7).

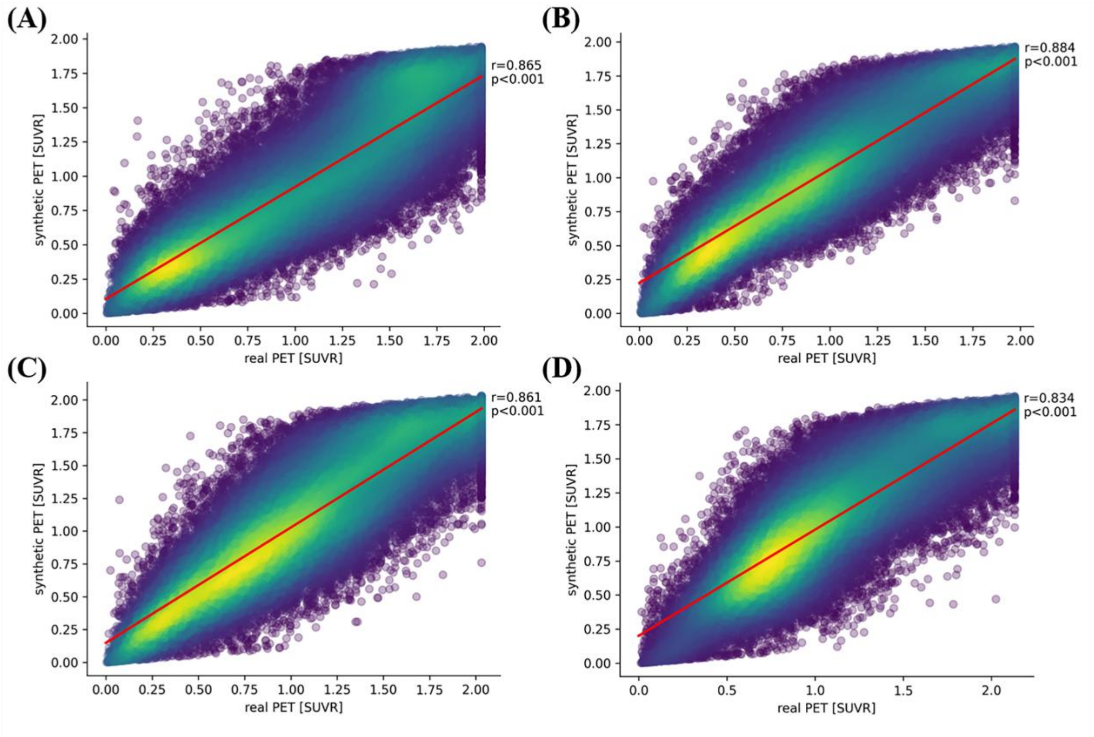

Fig S7: Representative scatter plots of voxel-wise correlation between synthetic PET and real PET in external testing set 1 and external testing set 2. (A) an AD patient (male, 59 years old, Aβ positive, education = 16 years, MMSE score = 4, APOE ε3/ε4) and (B) a HC participant (male, 67 years old, Aβ negative, education = 9 years, MMSE score = 29, APOE ε3/ε4) in external testing set 1. (C) an AD patient (female, 72 years old, Aβ positive, education = 9 years, MMSE score = 8, APOE ε3/ε4) and a HC participant (male, 73 years old, Aβ negative, education = 9 years, MMSE score = 28, APOE ε3/ε3) in external testing set 2.

8 Diagnostic Evaluation

The confusion matrices for diagnosis in each group based on 3D synthetic Aβ PET were presented in Table S2.

Table S2: Confusion matrix of 3D synthetic Aβ PET for dementia groups

Diagnosis based on real PET	Diagnosis based on synthetic PET	
	Positive	Negative
AD group		
Positive	139	5
Negative	/	/
MCI group		
Positive	24	0
Negative	11	30
HC group		
Positive	/	/
Negative	6	18
VaD group		
Positive	3	0
Negative	5	11
bvFTD group		
Positive	/	/
Negative	5	15
DLB group		
Positive	6	0
Negative	0	1
SD group		
Positive	/	/
Negative	2	6

Note: / means none of this parameter, because the Aβ status of bvFTD and SD is negative only.

Abbreviation: Aβ, amyloid-β; PET, positron emission tomography; AD, Alzheimer's disease; VaD, vascular dementia; bvFTD, behavioural variant frontotemporal dementia; DLB, dementia with Lewybodies; SD, semantic dementia.

Supplement material references

1 McKhann G, Drachman D, Folstein M, Katzman R, Price D, Stadlan EM (1984) Clinical diagnosis of Alzheimer's disease: report of the NINCDS-ADRDA Work Group under the auspices of Department of Health and Human Services Task Force on Alzheimer's Disease. Neurology 34:939-944. https://doi.org/10.1212/wnl.34.7.939.

2 Petersen RC (2004) Mild cognitive impairment as a diagnostic entity. J Intern Med 256:183-194. https://doi.org/10.1111/j.1365-2796.2004.01388.x.

3 Román GC, Tatemichi TK, Erkinjuntti T et al (1993) Vascular dementia: diagnostic criteria for research studies. Report of the NINDS-AIREN International Workshop. Neurology 43:250-260. https://doi.org/10.1212/wnl.43.2.250.

4 Harris JM, Gall C, Thompson JC et al (2013) Sensitivity and specificity of FTDC criteria for behavioral variant frontotemporal dementia. Neurology 80:1881-1887. https://doi.org/10.1212/WNL.0b013e318292a342.

5 McKeith IG, Boeve BF, Dickson DW et al (2017) Diagnosis and management of dementia with Lewy bodies: Fourth consensus report of the DLB Consortium. Neurology 89:88-100. https://doi.org/10.1212/wnl.0000000000004058.

6 Gorno-Tempini ML, Hillis AE, Weintraub S et al (2011) Classification of primary progressive aphasia and its variants. Neurology 76:1006-1014. https://doi.org/10.1212/WNL.0b013e31821103e6.

7 Pan X, Ge C, Lu R et al (2022) On the integration of self-attention and convolutionProceedings of the IEEE/CVF conference on computer vision and pattern recognition, pp 815-825

8 Mao X, Li Q, Xie H, Lau RY, Wang Z, Paul Smolley S (2017) Least squares generative adversarial networksProceedings of the IEEE international conference on computer vision, pp 2794-2802
